# Sprague-Dawley Rats Differ in Responses to Medial Perforant Path Paired Pulse and Tetanic Activation as a Function of Sex and Age

**DOI:** 10.1523/ENEURO.0431-22.2023

**Published:** 2023-07-04

**Authors:** Susan G. Walling, Carolyn W. Harley, Gerard M. Martin, Olivia D. E. Dutton, Alexander T. Burke, Ella A. Chirinos

**Affiliations:** Behavioural Neuroscience, Department of Psychology, Memorial University of Newfoundland and Labrador, St. John’s, Newfoundland and Labrador A1B 3X9, Canada

**Keywords:** age-dependent, entorhinal cortex, granule cell, long-term potentiation, sex differences, short-term plasticity

## Abstract

Network plasticity in the medial perforant path (MPP) of adult (five to nine months) and aged (18–20 months) urethane-anesthetized male and female Sprague Dawley rats was characterized. Paired pulses probed recurrent networks before and after a moderate tetanic protocol. Adult females exhibited greater EPSP-spike coupling suggesting greater intrinsic excitability than adult males. Aged rats did not differ in EPSP-spike coupling but aged females had larger spikes at high currents than males. Paired pulses suggested lower GABA-B inhibition in females. Absolute population spike (PS) measures were larger post-tetani in female rats than male rats. Relative population spike increases were greatest in adult males relative to females and to aged males. EPSP slope potentiation was detected with normalization in some post-tetanic intervals for all groups except aged males. Tetani shortened spike latency across groups. Tetani-associated NMDA-mediated burst depolarizations were larger for the first two trains in each tetanus in adult males than other groups. EPSP slopes over 30 min post-tetani predicted spike size in female rats but not in males. Replicating newer evidence MPP plasticity in adult males was mediated by increased intrinsic excitability. Female MPP plasticity was related to synaptic drive increases, not excitability increases. Aged male rats were deficient in MPP plasticity.

## Significance Statement

The medial perforant path (MPP)-dentate gyrus (DG) granule cell synapse was the site of discovery of long-term potentiation (LTP) in the mammalian nervous system but considering the current interest in sex and aging, surprisingly few studies have directly examined these variables in relation to tetanus-induced long-term and short-term (paired pulse) plasticity. Using an interleaved current-paired pulse interval protocol and moderate tetanic protocol young (five to nine months) and old (18–20 months) male and female urethane-anesthetized rats were found to differ in levels of granule cell intrinsic excitability, E-S coupling, GABA-B inhibition, and tetanic NMDA current contributions to post-tetanic population spike (PS) potentiation. This study provides a platform or future examination of sex-related and age-related changes in MPP-DG function.

## Introduction

In 1973, Bliss and Lomo published a landmark paper demonstrating tetanus-induced long-term potentiation (LTP) in medial perforant path (MPP)-evoked potentials recorded in the dentate gyrus of anesthetized rabbit (Bliss and Lomo, 1973). This paper became the starting point for hypotheses concerning the physiological basis of learning and memory. The enduring plasticity Bliss and Lomo found most frequently was reduced population spike (PS) latency, but increases in population EPSPs generated the greatest interest. Increases in PS amplitude were similarly frequent, but not consistently correlated with EPSP increases. Bliss and Lomo conclude that two independent mechanisms were responsible for MPP-LTP: (1) an increase in the efficacy of synaptic transmission and (2) an increase in the excitability of the granule cell population. The preponderance of experimental LTP investigations in dentate gyrus focused on the (1) mechanism, EPSP potentiation. However, in the last two decades, attention has turned to excitability increases. In 2016, Lopez-Rojas et al. (2016) presented evidence that an increase in dendritic intrinsic excitability is primarily responsible for dentate gyrus MPP-LTP in mature granule cells of male rats. A comparative review of EPSP and intrinsic excitability changes in learning and memory (Daoudal and Debanne, 2003) highlights the commonality of their induction mechanisms with both depending on NMDA receptors. Further, both types of plasticity events may act bi-directionally, increasing or decreasing connectivity, in neural networks. Sex and age differences have been understudied in MPP plasticity. The present set of experiments address those variables in the context of Bliss and Lomo’s original observations.

## Materials and Methods

Male and female Sprague Dawley rats (Charles River) were housed doubly in individually ventilated cages (Techniplast) with regular enrichment, on a reversed light cycle 12/12 h cycle (lights off at 7 A.M.) until the age of approximately two to four months, and then singly housed thereafter. Rats were fed regular chow (Teklad2018); however, both male and female rats were placed on a modest food restriction schedule at two to three months of age, to maintain a healthy aging weight profile and to reduce obesity related illnesses in old age (Hubert et al., 2000). Rats were fed between 8:30 and 10:30 A.M. daily and received an amount of food that was 75% (g) of the average age-dependent and sex-dependent *ad libitum* consumption (Hubert et al., 2000). Water was available *ad libitum*. The average mass at the time of electrophysiological recording in the five- to nine-month-old rats; females 366.33 ± 27.45 g and males 674.8 ± 38.89 g, and for 18- to 20-month-old rats; females 456.00 ± 81.82 g and males, 748.80 ± 71.11 g.

All experimental procedures occurred within the dark phase of the animals’ light cycle (9 A.M. to 5 P.M.) and were performed in accordance with the Canadian Council of Animal Care (CCAC) guidelines and approved by the Memorial University of Newfoundland and Labrador Institutional Animal Care Committee.

### Electrophysiological recording

**Figure 1.**
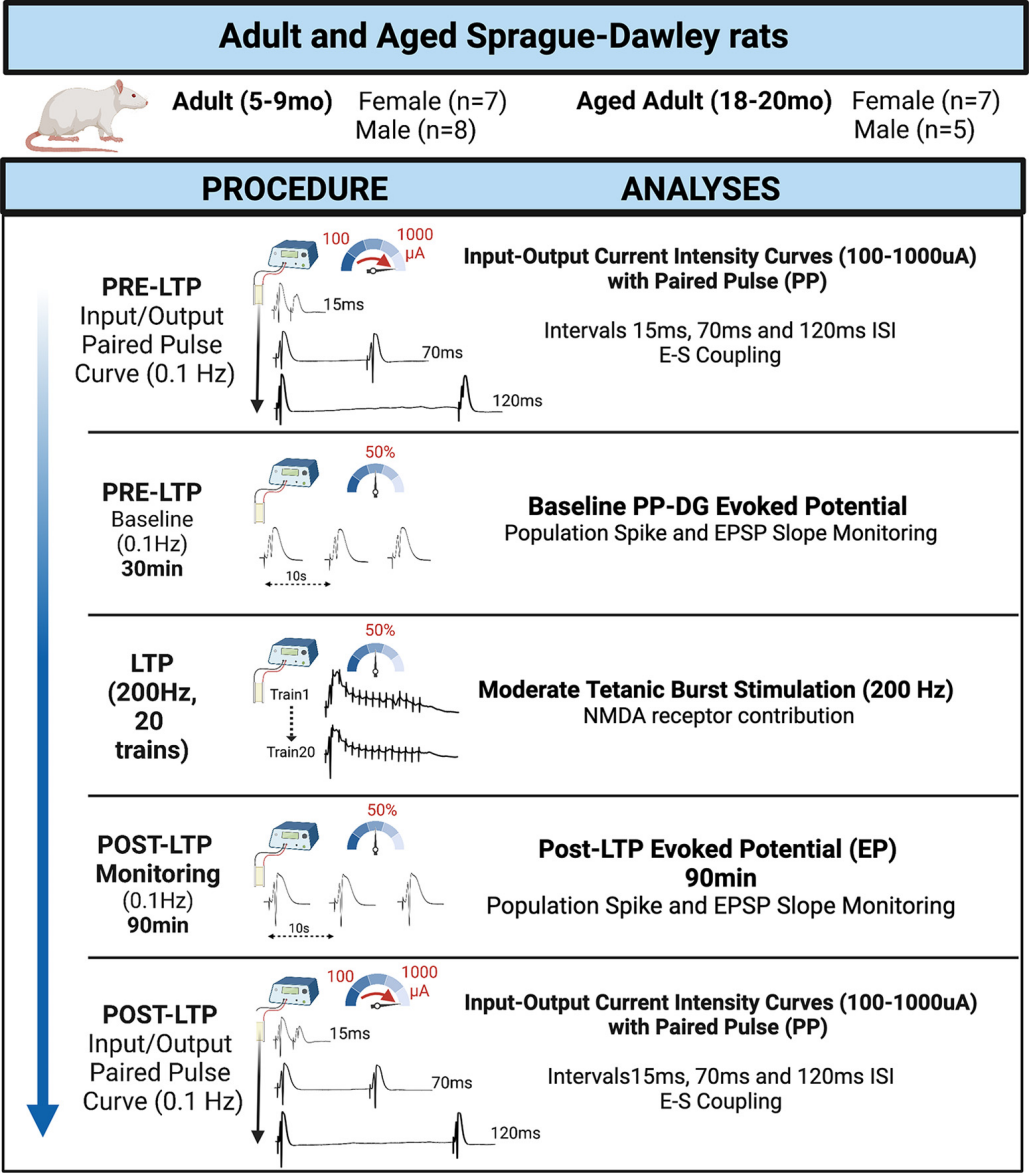
Graphical experimental procedure. In brief, experiment commenced with paired pulse (15, 70, and 120 ms interleaved on a current intensity profile; 100–1000 μA). Baseline recording (min 30 min) with current eliciting 50% maximal PS. Moderate strength tetanic stimulation (Protocol B, Straube and Frey, 2003), followed by 90-min recording (0.1 Hz). Post-LTP PP and input-output current intensity curve. Image BioRender.

At 5–9 or 18–20 months of age, rats were anesthetized with urethane (intraperitoneally). To reduce overdose susceptibility because of age, sex, or food restriction (Davis et al., 2000), rats were stepped up to a ∼1.5 g/kg dose (15% w/v). Once anesthetized the rats were placed in a stereotaxic instrument in the skull flat position and body temperature was maintained at 37 ± 0.5°C via a feedback-regulated heating pad (FHC). A concentric bipolar stimulating electrode (NE-100; Kopf) was lowered into the PP (∼7.2 ± 0.3 mm posterior, and ∼4.1 ± 0.1 mm lateral to bregma, and ∼3.0 mm ventral from brain surface) and a glass pipette (0.9% NaCl, 1–3 MΩ) was lowered into the DG (∼3.5 ± 0.2 mm posterior and ∼2.0 ± 0.2 mm lateral, and ∼2.5 mm ventral; adjusted for animal size). A stainless-steel jeweler’s screw (Fine Science Tools) served as ground. A 0.2-ms square unipolar test pulse was delivered (0.1 Hz) to the PP and the DG responses via a constant current stimulation unit (NeuroData Instruments) were amplified and filtered 1–10 kHz (Grass Instruments), digitized at 10 kHz, and stored online using SciWorks 9.0 or 11.0 software (Datawave Technologies). The stimulating and recording electrodes were then adjusted in the dorsal/ventral plane so that a maximal positive going waveform (granule cell layer) was achieved. See graphical experimental procedures in Figure 1 for outline of experimental procedures and analyses.

### Current intensity E-S coupling and paired pulse analyses

At the commencement of the experiment, an input-output (I-O) current intensity curve (100–1000 μA, 100-μA steps) was performed using a series of three paired pulses presented every 10 s at each current level. Sets of paired pulses consisted of one presentation each of interstimulus intervals (ISIs) 15, 70, and 120 ms such that each current level tested each ISI before the current was increased. Similar I-O and E-S profiles were reported in Walling and Harley (2004) in awake male rats using I-O current stimulation (0.1 Hz; 50–1000 μA) without paired pulse assays suggesting that paired pulse procedures did not alter I-O or E-S relationships. The paired pulse intervals were chosen to probe GABA-A sensitive paired pulse inhibition (PPI; 15 ms), paired pulse facilitation (PPF; 70 ms) and GABA-B late paired pulse inhibition (PPI; 120 ms) of the PP-DG evoked PS in urethane-anesthetized male (Joy and Albertson, 1993; Ruan et al., 1998) and female (Brucato et al., 1992) rats. The current level used for baseline stimulation during the PP-DG LTP experiment was the current producing a PS on the first (P1) of the two evoked paired responses that was ∼50% of maximum PS amplitude achieved in the I-O current curve. These current-paired pulse procedures were performed again at the termination of the LTP recording procedures to determine the effect of LTP on current intensity analysis and paired pulse facilitation and inhibition.

I-O current E-S coupling analysis was performed by averaging the three first pulse PS and EPSP slope measurements at each current level in the pre-LTP and post-LTP curves. These were then contrasted across a range of EPSP measures (low current, smaller EPSP slope; through higher current and larger measures of the synaptic response). Measures are presented as absolute values (mV/ms and mV for slope and PS, respectively) and normalized to the largest, average PS and EPSP slope from the pre-LTP current curve (Walling and Harley, 2004).

Paired pulse ratios (PPR, pulse2/pulse1; P2/P1) were calculated for the PS amplitude at each ISI, and at each current intensity level. For presentation, the P2/P1 ratio from the stimulation intensity used for the LTP experiment (baseline; ∼50% maximal PS) was contrasted with P2/P1 ratio at the same current intensity after LTP (post-LTP). For PPR current intensity analyses (Extended Data Fig. 5-1) outlier PPR values, in which the population spike amplitude on the second pulse was >3 SDs above normal, PPR values were capped to +2 SD of the highest PPR value for the animal at the respective ISIs. This treatment was applied to 4/360 PPR values in the six- to eight-month-old male rats and 3/360 values in 18- to 20-month-old female rats.

### Moderate strength burst long-term potentiation (LTP) stimulation

After completion of the initial I-O and paired pulse curves, a tetanic LTP protocol was tested. This protocol of the three tested by Straube and Frey (2003), the moderate Protocol B, produced robust, β-adrenergic receptor, and protein synthesis-dependent, LTP of the PS variable in behaving male Wistar rats. The PP was stimulated at 0.1 Hz with a monophasic 0.2-ms pulse and after 30 min of stable baseline evoked responses, monophasic tetanic stimulation at 200 Hz was applied, consisting of 20 trains of 15 pulses (0.25-ms width) with 10-s interburst intervals. Following the tetanic stimulation, the PP-DG evoked responses were followed for 90 min recording (0.1 Hz) followed by the second I-O and paired pulse analysis. Conventional absolute and normalized baseline and post-tetanic EPSP slope and PS measures are presented in Extended Data Figure 2-1.

#### Assessment of the synaptic contribution to PS plasticity in adult and aged male and female rats

To assess the contribution of synaptic input on enduring PS potentiation, absolute measures of the EPSP slope and PS were arranged in 30-min bins and the EPSP slope measure of the first post-tetanic 30-min bin (0–30 min) was correlated (Pearson) with the three 30-min post-tetanic PS measures (0–30, 31–60, and 61–90 min post-LTP). For contrast, the EPSP slope and PS measures are also presented from the 30-min baseline period to illustrate pretetanus correlations.

##### Analysis of indexed NMDA receptor current during moderate strength tetanic LTP

To assess differences in NMDA receptor activation during tetanic in adult and aged adult male and female rats, total area under the curve (AUC) was measured first for each pulse within a 15 pulse stimulation train for each of the 20 burst stimulations, beginning at ∼10 ms after the first pulse, a period determined previously to constitute NMDA receptor activation (Racine et al., 1991; Maren et al., 1992). Total NMDA AUC was also summed across all 20 trains and also contrasted with the post-tetanic PS potentiation (see Extended Data Fig. 4-1).

### Statistical analysis

With the exception of correlative variables, data were analyzed using multifactor analysis of variance (baseline EPSP slope and PS) or mixed design analysis of variance (age, sex, variable). Tukey’s HSD test was used for *post hoc* assessments. All analyses were performed using Statistica v13.5 (StatSoft).

## Results

### Female rats show greater intrinsic granule cell excitability than males both pre-LTP and post-LTP. In adult male rats the LTP protocol induces an increase in excitability but does not induce an excitability increase in female rats

**Figure 2.**
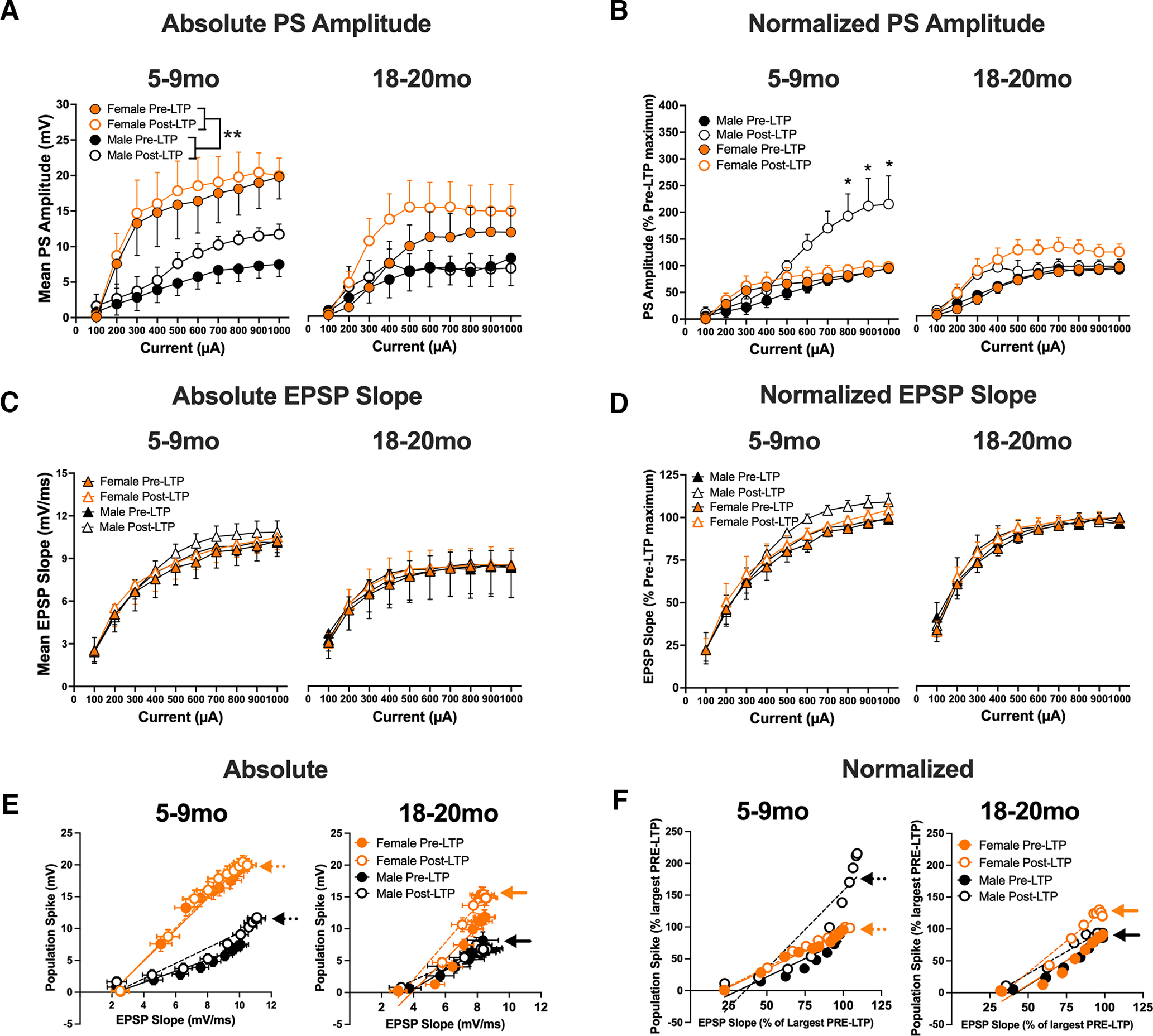
The effects of a moderate strength tetanic LTP protocol on PS and EPSP slope current intensity curves (input-output, I-O) and E-S coupling of the medial perforant path input to dentate gyrus in adult and aged adult, male and female rats. ***A***, Absolute PS I-O values for adult and aged adult male and female rats pre-LTP and post-LTP. A main effect of sex was revealed (*F*_(1,23)_ = 7.35; *p* = 0.01). ***B***, A significant sex, age, current, LTP interaction was found when PS values were normalized (*F*_(9,207)_ = 3.27; *p* = 0.0009). ***C***, Absolute EPSP slope I-O analysis. ***D***, Normalized EPSP slope I-O analysis. ***E***, E-S coupling. Mean absolute EPSP slope (*x*-axis) and PS measures (*y*-axis) plotted at each current intensity (100–1000 μA). EPSP and PS values in adult (5–9 months) rats indicate a leftward shift in E-S coupling values in female rats, and values indicative of a ceiling effect post-LTP (orange dashed arrow). In male rats at both ages PS values are ∼40–50% of female PS values (black vs orange arrows). ***F***, Relative E-S plot with data normalized to the largest average PS and EPSP slope values from the Pre-LTP I-O curve. Aged female rats expressed larger PS increases post-LTP but retained similar pre-LTP and post-LTP EPSP slope values, suggesting higher levels of intrinsic excitability support post-tetanic potentiation of the MPP input to DG (solid orange arrow). Aged male rats failed to express enduring potentiation of either EPSP slope or PS measures. Data represent means ± SEM; however, error bars in ***F*** cannot not be observed because of the small levels of variability. *p < 0.05, **p < 0.01. Sample waveforms and electrode placements, and conventional temporal LTP analyses of EPSP Slope and PS variables (absolute and normalized) are provided in Extended Data Figure 2-1.

The involvement of intrinsic excitability in MPP LTP in adult male rats is consistent with the findings of Lopez-Rojas (2016). The greater normalized LTP in adult male than adult female rats (Fig. 2; Extended Data Fig. 2-1*C*) replicates Maren (Maren et al., 1994; Maren, 1995). Higher levels of excitability in the MPP circuit of female rats than male rats are seen in datasets from earlier studies (Kehoe and Bronzino, 1999; Zitman and Richter-Levin, 2013; Safari et al., 2021; raw data shared by Safari et al., 2021), but see Elmarzouki et al. (2014) for counter example.

10.1523/ENEURO.0431-22.2023.f2-1 Extended Data Figure 2-1 The effects of a moderate strength tetanic LTP protocol on the perforant path-dentate gyrus evoked population spike (PS) and EPSP slope measures in adult (5–9 months) and aged adult (18–20 months) urethane-anesthetized male and female rats. ***A***, Sample dentate gyrus recording electrode placements and MPP-dentate gyrus evoked potential waveforms for three periods before (baseline) and after (0 and 90 min) moderate strength tetanic stimulation. Scale bar is 500 μm in micrograph and 4 mV and 5 ms in waveforms. ***B***, Absolute PS amplitude (baseline current level), PS latency (baseline, 0 min, and 90 min post-LTP) and EPSP slope measures (baseline current level). PS values (mV) were significantly higher in female rats compared to male rats at baseline current levels (first graph). PS latency decreased similarly post-LTP for all groups (middle graph). Absolute EPSP slope values did not differ between groups at baseline current intensity (third graph). ***C***, Temporal profile (X-Y plot), and 30-min binned data (bar graph) of absolute (top panels), and normalized (bottom panels) PS data. Female rats had significantly larger PS (mV) amplitude measures than male rats (main effect sex, *F*_(1,23)_ = 7.378; *p* = 0.012). Normalization of PS data illustrates adult males (5–9 months) had higher percentage PS increases than aged males (18–20 months), and adult female rats (age, sex, LTP interaction, *F*_(3,69)_ = 6.52; *p* = 0.0006, with *post hoc*). ***D***, EPSP Slope data. No sex-dependent or age-dependent differences were observed in absolute EPSP slope measures (top panels). When post-LTP EPSP slope values were normalized to baseline measures; however, a significant age × sex interaction was also revealed (*F*_(1,23)_ = 5.703; *p* = 0.026). Normalized baseline measures were not included in the statistical analyses in ***C*** or ***D*** (gray bars). * minimum *p* < 0.05, **p < 0.01. Download Figure 2-1, TIF file.

Adult (five- to nine-month) female rats have a leftward shift in E-S coupling relative to same age males, however PS increases in adult females appears to reach a reach a ceiling effect (Fig. 2*A*,*E*), while absolute measures of PS amplitude in males are ∼40–50% of female PS values in both adult and aged adult rats. Aged males and females have a similar E-S coupling EPSP slopes while aged female PS values increased post-LTP from pre-LTP values, a characteristic not observed in aged male rats. This argues that higher female intrinsic excitability, while possibly diminished in aged females from that of five- to nine-month female values, is still present.

#### Increases in the population spike post-LTP in female rats are most predicted by early (first 30 min post-LTP) increases in EPSP slope

**Figure 3.**
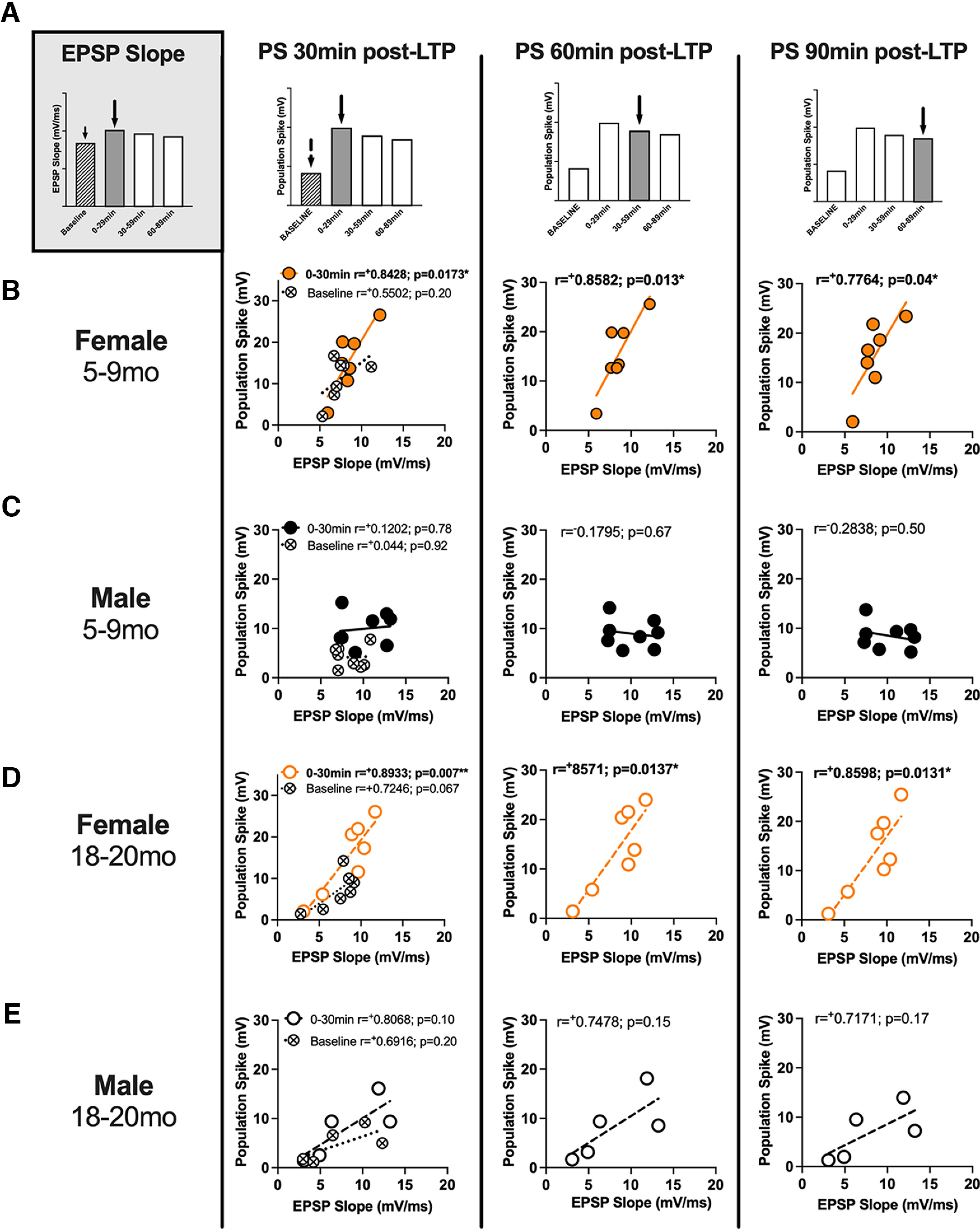
The effects of a moderate strength tetanic LTP protocol on the perforant path-dentate gyrus evoked population spike in young (5–9 months) and old (18–20 months) urethane-anesthetized female rats, correlate with early (0–30 min post-LTP) EPSP slope potentiation. ***A***, Absolute EPSP slope and PS values were organized into 30-min bins (baseline, 0–30, 30–60, 60–90 min post-LTP). EPSP slope values for the baseline period, and 0–30 min post-LTP (early increases) were plotted against the baseline and the 0- to 90-min PS binned values. Baseline correlations indicate that EPSP slope is not significantly correlated with PS values in any sex, or age group (crossed circles, ***B–E***); however, post-LTP EPSP slope values are predictive of increases in PS post-LTP in female rats (adult and aged; see bolded correlations in ***B*** and ***D***). This effect was not observed in adult, or aged adult male rats (***C*** and ***E***). Additional comparisons of EPSP slope and PS measures at matched time bins (30–60 and 60–90 min) are provided in Extended Data Figure 3-1.

In female rats, the PS-LTP following tetani is predicted by the EPSP slope increase occurring in the first 30 min post-tetani (Fig. 3). This post-tetani EPSP-spike correlation is not significant for male rats. When later post-LTP EPSP slope epochs (30–60 and 60–90 min) were examined to contrast with the results of the early EPSP changes, the EPSP slope measures were again, most predictive of PS values in female but not male rats of either age, however the latest (60–90 min) epoch in five- to nine-month female rats no longer reached significance indicating the early EPSP changes may be most predictive of PS amplitude changes over time; see Extended Data Figure 3-1. Aged males also fail to show significant normalized slope or spike increases following tetani (see Fig. 2; Extended Data Fig. 2-1). These outcomes argue that of the two MPP plasticity mechanisms identified by Bliss and Lomo (1973), an increase in synaptic size drives an increase in population spike in dentate gyrus of female rats, while in adult male rats plasticity depends on an increase in granule cell dendritic excitability as shown by Lopez-Rojas (2016). All groups showed a decrease in spike latency following tetani (Extended Data Fig. 2-1). This outcome is consistent with Bliss and Lomo’s report that spike latency decrease was the most consistent response to LTP protocols (Bliss and Lomo, 1973). In a new study of MPP EPSP slope potentiation, Amani et al. (2021) report a decline in normalized EPSP potentiation beginning as early as eight months in male rats. The present failure of older males to exhibit EPSP potentiation corroborates their finding.

10.1523/ENEURO.0431-22.2023.f3-1 Extended Data Figure 3-1 The effects of a moderate strength tetanic LTP protocol on the perforant path-dentate gyrus evoked population spike in young (5–9 months) and old (18–20 months) urethane-anesthetized male and female rats examined at matched 30- to 59- and 60- to 90-min time periods. Similar to results examining the correlation between early (0–30 min) post-LTP EPSP slope potentiation on population spike potentiation at 0–30, 30–60, and 60–90 min post-LTP periods (presented in Fig. 3), the EPSP slope potentiation of female rats (adult and aged adult) was still most often a predictor of PS potentiation; however in contrast to the early EPSP potentiation (Fig. 3), late (60–90 min) EPSP slope potentiation did not significantly correlate with PS potentiation in adult (5–9 months) females. Male rat EPSP slope potentiation (early or late) did not correlate with PS potentiation at any of the post-LTP periods. Download Figure 3-1, TIF file.

### NMDA burst activation was stronger in adult males than in adult females or aged males

**Figure 4.**
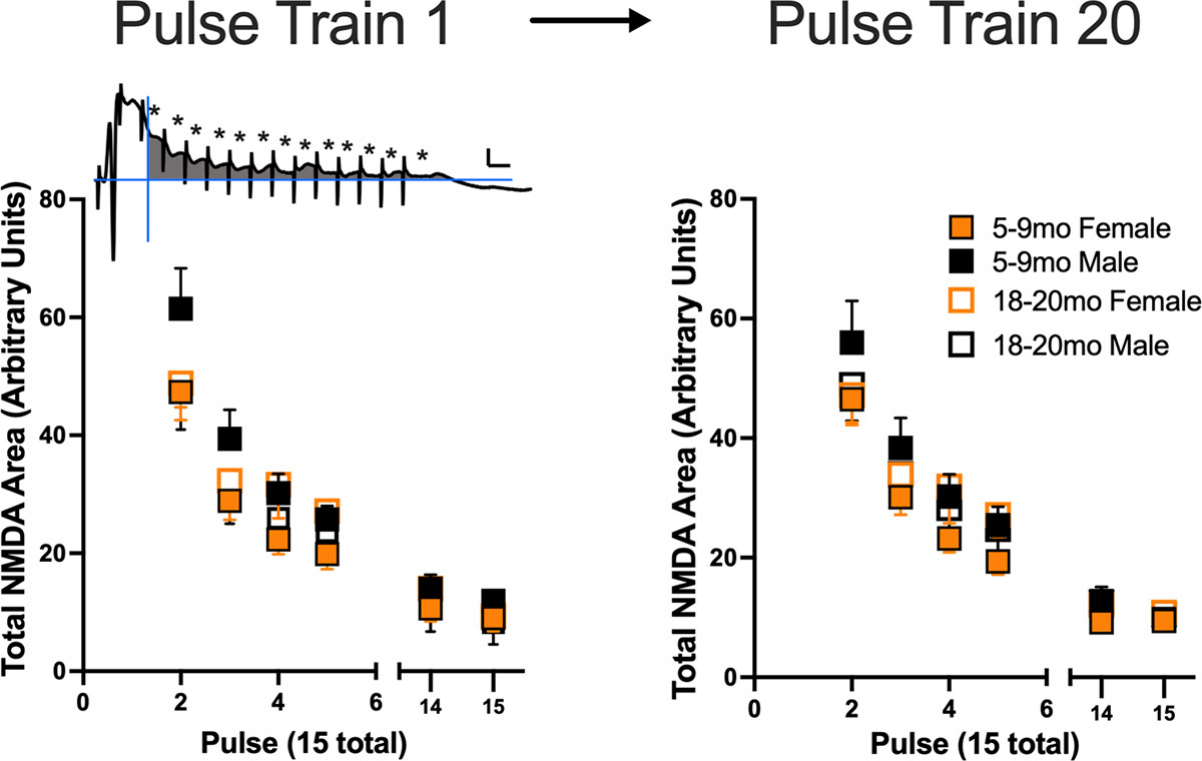
Assessment of the total NMDA receptor depolarization (Maren et al., 1992) for the first three trains during tetanic stimulation. The male adult rats (5–9 months, left panel) had a larger NMDA-dependent component than adult female rats (*F*_(1,13)_ = 5.1936; *p* = 0.04). In aged adult rats (right panel), there were no sex-dependent differences observed. In both age groups, there was a significant effect of trial with successive trains decreasing in the total NMDA-dependent response to the tetanic stimulation rats (adult, *F*_(2,26)_ = 4.1925; *p* = 0.02; aged adult, *F*_(2,20)_ = 11.567; *p* = 0.0005). Scale is 4 mV/5 ms. Total NMDA receptor contribution during moderate tetanic LTP stimulation correlated with PS increase in 30-min bins in adult and aged male and female rats (see Extended Data Fig. 4-1).

Using Maren’s approach (Maren et al., 1992) of examining postburst depolarization to evaluate NMDA activation by tetani, we found the first three trains provided evidence of greater adult male (five to nine months) NMDA burst depolarization than that seen in female rats of the same age (Fig. 4). Consistent with this finding, Maren et al. (1992) reported larger NMDA burst depolarization in adult males than adult females. NMDA burst depolarization here decreased successive trains across the sexes, in both age groups. The outcome suggests NMDA induction is weakest in adult females, but not aged females. Effects of total NMDA AUC for the 15 pulses across the 20 trains was more predictive of PS potentiation in aged rats (see Extended Data Fig. 4-1).

10.1523/ENEURO.0431-22.2023.f4-1 Extended Data Figure 4-1 **Total NMDA receptor contribution during moderate tetanic LTP stimulation correlated with population spike increase in 30min bins in adult and aged male and female rats.** The total NMDA receptor Area Under the Curve (AUC) for the 15 pulse, and 20 tetanic trains was plotted against the absolute PS amplitude (mV) for the post-tetanic period (0-90min post-LTP). Total NMDA AUC was not correlated with absolute PS values (A-B) however, a significant correlation was more associated with aged male (18-20mo, see E), and trend in aged female rats (D). Download Figure 4-1, TIF file.

### Paired pulse data revealed less GABA-B inhibitory modulation in female than male rats

**Figure 5.**
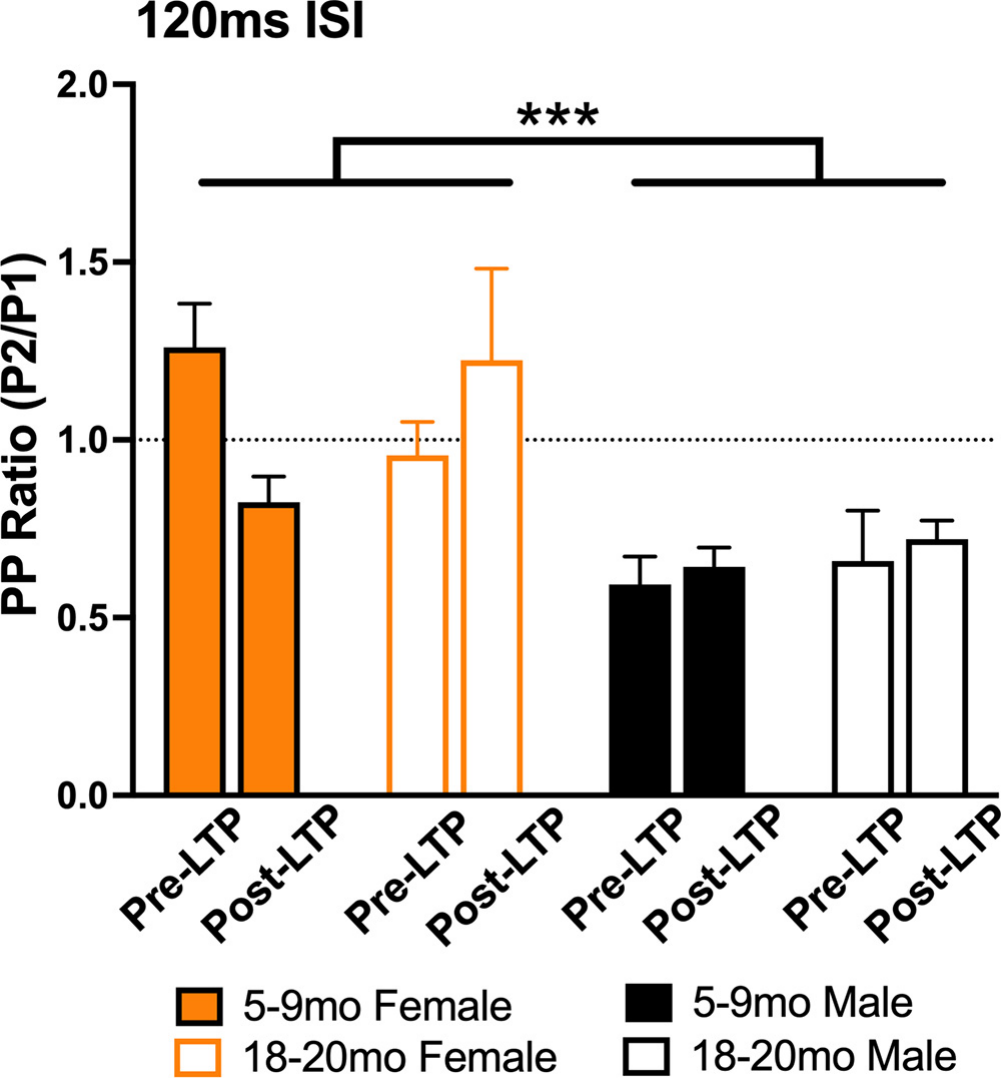
Paired pulse inhibition (120-ms ISI) and facilitation of the PP-DG evoked population spike in young and old male and female rats. P2/P1 ratio for GABA-B dependent PPI (in 5- to 9- and 18- to 20-month male and female rats at current levels used for baseline recordings; 50% maximal PS on P1), and same current post-LTP. Female rats demonstrated less PPI compared with male rats. There was a significant main effect of sex (*F*_(1,23)_ = 17.49; *p* = 0.0003). Data represent means ± SEM, ***p < 0.001. See Extended Data Figure 5-1 for full description of PP input-output profiles.

Paired pulse inhibition probed at a 120-ms interval revealed the expected inhibition in male rats when the P1/P2 ratio was summed over all currents (∼50% inhibition; see Fig. 5). Females, however, did not show evidence of 120-ms ISI inhibition with their P2/P1 ratios averaging close to or above 1.0. Reduced GABA-B-mediated inhibition, indexed by this probe, may contribute to greater female intrinsic excitability. Canning and Leung (2000) demonstrated that *in vivo* granule cell excitability is controlled by GABA-B-mediated inhibition. See Extended Data Figure 5-1 for full profile of input-output current curve paired pulse results.

10.1523/ENEURO.0431-22.2023.f5-1 Extended Data Figure 5-1 The effects of a moderate tetanic LTP protocol on paired pulse ratio input-output current intensity curves for male and female adult and aged adult Sprague Dawley rats at three interstimulus intervals. ***A***, 15-ms ISI (PPI); no significant main effects or interactions of sex for either age groups were observed. ***B***, 70-ms ISI (NMDA-sensitive PPF; Joy and Albertson, 1993) no significant main effects or interactions of sex for either age groups, however examples of hyperexcitability emerged in some male and female rats (examples shown, arrows in waveforms). These could not be quantified. ***C***, 120-ms ISI (PPI). In the five- to nine-month age group, there was a significant main effect of Age (*F*_(1,13)_ = 18.46; *p* < 0.001), and in the 18- to 20-month rats, a significant current × sex interaction (*F*_(9,90)_ = 3.106; *p* = 0.003). Burdette and Gilbert (1995) report late PPI at low current levels (200-ms ISI) in behaving male Long–Evans rats similar to the urethane-anesthetized adult (5–9 months) male and female and aged male Sprague Dawley rats here; however, this profile was less observed in aged adult (18–20 months) female rats in the present study. Data represent means ± SEM; **p < 0.01 and ***p < 0.001. Scale in waveforms is 4 mV/20 ms. Download Figure 5-1, TIF file.

## Discussion

The present experiments provide evidence for both sex-related and age-related differences in MPP plasticity in rat dentate gyrus. The sex difference in intrinsic excitability requires replication. While re-examining control data in earlier studies with other objectives provides some support for our findings, only one other laboratory has specifically addressed this variable. Maren’s studies did find greater normalized spike potentiation among Sprague Dawley males under two kinds of anesthesia, but they specifically matched male and female rats for spike size potentially eliminating the differences in absolute spike/current relationships seen here.

What sex differences might account for these outcomes? Erwin et al. (2020) demonstrate that a subtype of mature granule cell, the PENK-expressing granule cell is more excitable than other subtypes and because of its greater excitability is preferentially recruited to spatial maps. They argue that this subtype dominates recruitment in hippocampally-dependent behaviors and supports sparse spatial representations in male mice. The critical role of intrinsic excitability in determining dentate gyrus MPP output was also recently highlighted by Zhang et al. (2020). Oulé et al. (2021) identified the potassium channels responsible for dendritic intrinsic excitability in male mice as the Kv4.2 subtype and revealed that these channels are critical for spatial pattern separation.

In a new study identifying PENK-expressing granule cells in both male and female rats, Johnson et al. (2021) demonstrate that female dorsal dentate gyrus contains a significantly larger proportion of PENK-expressing granule cells than male dentate gyrus. The higher PENK-cell proportion in females may support their greater intrinsic excitability. Based on the data from mice, MPP-supported spatial and nonspatial representations might be predicted to be less diffusely distributed, and therefore possibly more robust, in female rats than males (see, for example, Zhvania et al., 2021; Olave et al., 2022; O’Leary et al., 2022). Experimental evidence with respect to place field and episodic representation in female versus male rats is needed. In an evolutionary context (Sherry et al., 1992) spatial map demands appear different for male and female rats. Male *Rattus norwegicus* rats have spatial territories an order of magnitude larger than females (Oyedele et al., 2015) and may require better spatial resolution for those territories.

The role of aging in dentate gyrus plasticity has been more extensively studied. A selective decrease in MPP synapses on granule cells dendrites (3 vs 28 months) was reported in 1976 (Bondareff and Geinisman, 1976). Barnes (1979) comparing behavioral and LTP measures in 10- to 16- versus 28- to 34-month-old male rats found repeated trains produced enduring EPSP slope potentiation at 10–16 months but a declining potentiation in the senescent rats. Barnes et al. (2000) report reduced NMDA currents and a higher threshold for EPSP LTP in senescent rats, which were memory-impaired (see also Yang et al., 2008). Their middle-aged group (9–10 months), are similar to the adult rats here and appeared intermediate between young and senescent rats in depolarization needed for EPSP slope potentiation. In the Barnes and colleagues study, the NMDA currents in female rats were not examined and warrant further investigation. Arc-active granule cells with spatial exploration decline across young, middle and aged rats (Small et al., 2004).

The state of estrus or levels of circulating hormones were not monitored in this study. Gould et al. (1990) examined dendritic spine density changes in the hippocampus across the estrus cycle and found density changes in CA1, but did not find that estrus affected spine density in dentate gyrus granule cells. Kehoe and Bronzino (1999) similarly did not find differences in dentate gyrus LTP across the estrus cycle in awake female rats (three-month-old rats). Together, this suggests the state of estrus in the five- to nine-month female rats was not a significant influence on plasticity. It would be presumed that the 18- to 20-month female rats had low levels of brain and circulating estrogen, however aged females still expressed higher levels of PS plasticity compared with aged male rats. Influences of estrogen across the lifespan may still account for these differences potentially providing a plasticity “reserve” in aged females. Sex-dependent differences in neurogenesis in dentate gyrus during aging may similarly explain the changes in dentate gyrus plasticity (Yagi et al., 2020; Hodges et al., 2022). Given that MPP-dentate gyrus plasticity in aged females is remarkably spared compared with age-matched male rats in the present study, investigations into the possible neuroprotective role of estrogen, and influence on neurogenesis during aging should be comprehensively examined.

Multiple laboratories (Rapp and Gallagher, 1996; Lubec et al., 2019) have demonstrated both memory-impaired and memory-unimpaired aged male rats are seen when probed on hippocampally-dependent tasks, thus variability in initiating and maintaining plasticity with age is likely. Informal examples of age-related and sex-related heterogeneity of dentate gyrus plasticity can be observed in variability measures in long-term plasticity (see Extended Data Fig. 2-1) and short-term plasticity (see aged female rats in Extended Data Fig. 5-1 at low stimulation levels). Future studies could further examine heterogeneity of plasticity measures related to age and sex and its possible relationship with behavior. Understanding the mechanistic underpinning of that variability will be useful for cognitive anti-aging strategies. Lubec et al. (2019) working with Sprague Dawley rats aged 22–24 months and comparing them to six-month rats identified proteomic changes that occurred differentially in aged impaired and unimpaired rats. Impaired rats were deficient in pathways related to energy metabolism and potassium ion regulation. They found unimpaired rats differed from the general population as early as six months of age corroborating the Amani et al. (2021) assertion that decreases in MPP plasticity are an early harbinger of aging. Lubec et al. (2021) later found that increasing dopamine in aged male Sprague Dawley rats with both intermediate and severe behavioral impairments on hippocampally-dependent tasks restored behavior to that of young rats and restored spine numbers on granule cells to young levels. Both spatial behavior and cognitive flexibility were improved.

The present experiments suggest MPP-related aging changes are likely to differ among males and females with males being more vulnerable to aging-related disruption of plasticity and likely to show greater impairment on hippocampally-dependent tasks. This hypothesis remains to be explored.

## Synthesis

Reviewing Editor: Matthew Grubb, King’s College London Decisions are customarily a result of the Reviewing Editor and the peer reviewers coming together and discussing their recommendations until a consensus is reached. When revisions are invited, a fact-based synthesis statement explaining their decision and outlining what is needed to prepare a revision will be listed below. The following reviewer(s) agreed to reveal their identity: Jeffrey Lopez-Rojas.

The manuscript has been seen by two reviewers. Both agree that the manuscript advances the field and is important. However, the reviewers have made several comments and suggestions which need to be addressed before the manuscript can be suitable for publication.

Reviewer 1

The manuscript seeks to understand differences in plasticity at the medial perforant path-Dentate Gyrus synapses among sex and aging in rats. Changes in excitability, rather than the more classical synaptic changes, are reported by the authors in response to a relatively mild stimulation protocol. The authors describe those changes in adult and aged males and females and investigate possible mechanisms for such differences. Changes in excitability are relatively poorly understood compared to synaptic plasticity. However, neural excitability contribution to learning, memory and pathological conditions is critical. The manuscript is, in general, well-written and follows a logical argument. The results are exciting. However, I have some comments:

- It is not convincing to me to correlate the initial level of EPSP potentiation with the PS potentiation at all three time points considered (Figure 3). In my opinion, just the correlation for PS-EPSP baseline and PS-EPSP for the first 30min should be shown. If the 60min or 90min are shown, then the correlation should be done between the PS and EPSP corresponding to those timepoints.

- The heading “Increases in the population spike post-LTP in female rats depend on increases in EPSP slope” is misleading. According to Extended Data Figure 2-1, EPSPs are only transiently potentiated in all cases. Thus, EPSP potentiation cannot explain PS changes observed in aged females.

- Even though their information is implicitly shown in Fig.2, showing the I-O curves of PS and EPSP vs. Stimulation Intensity would help. Also, error bars should be displayed in Figure 2. In this regard, even though at the test stimulation intensity used for baseline and subsequent recordings after tetanic stimulation (50% of maximum PS) no EPSP potentiation was shown (Extended Data Figure 2-1), it seems from some of the graphs (Figure 2) that EPSP values were larger post-tetanus than in baseline in some cases (for instance in Figure 2A5-9mo). However, I have also some concerns about the I-O curves generated with the paired pulses every 10 secs and how this relatively short inter-stimulation interval and the paired pulses might have some effects on the I-O P1 values.

- The explanations are, in some instances, difficult to follow. For example, in the second paragraph of the Results section, it might help to include the particular graph the authors are referring to (i.e., PS values in aged males are ∼50% of female PS values (Figure 2A 18-20mo)).

- Part of the Extended Data Figure 2-1 legend seems missing. It is not clear to me what is shown in panel B.

- Using Fisher LSD as a post-hoc test might have some drawbacks as it usually does not account for multiple comparisons.

- The y-axis label on Figure 2B18-20mo should be “Population Spike (% of baseline)”.

- How frequent was the test stimulation during baseline and following tetanic stimulation?

Reviewer 2

Beautiful comprehensive in vivo paper of short- and long-term plasticity measures in adult rats. Elegant study design and large consistent field potential recordings. Topic areas has not been systematically addressed despite the significance of the model since its discovery and report over 50 years ago and the impact that discovery has had on the field. The analysis in this report is thorough and data reporting in main and supplemental materials, although dense, the graphics are complex but clear and complete. Text on figures is a little on the small side in several of the figures.

Some information on the software/hardware used to collect electrophysiological data could be included.

I did not see Extended Figures 2 or 3 in the materials. Extended Figure 1 was not labelled as such but I assume Temporal Profile is Extended Figure 1. Is there an Extended Figure 3 as the next Item listed is Extended Figure 4.

Modulating factors pertinent to the inquiry of sex and age differences that seem pertinent to the topic area but were not addressed by the authors include estrogen/estrous cycle in younger females and age-dependent differences in dentate gyrus neurogenesis.

The final paragraph concludes that males may be more vulnerable to the aging-related disruption of plasticity. Although not investigated here it seems that some speculation could be offered by the authors on the role of estrogen in offered some degree of protection to females.

As noted by the authors, age-dependent behavioral impairments reported by work of Gallagher demonstrates a biomodal distribution of impaired and non-impaired aged rats. The variability observed in Extended Figure Temporal Profile of the present study certainly suggests a nonhomogeneous populations as well for electrophysiological attributes, especially for the EPSP (Figure B). Similarly, in the Extended Material Figure 5-1 could the more variable responses to old females at all lSI at the lower stimulus intensities, most notably post LTP, also reflect heterogeneity in the aging profile?

Other Points

Curious to know if the authors experienced any additional difficulty with placement in the steretotaxic to attain a flat skull and consistency of electrode placement in the older animals relative to the young. Thicker skulls and alterations in toothbar angles can make the older animals more challenging. Perhaps this was mitigated somewhat from food restriction imposed.

It is clear from waveform morphology at the appropriate sites were hit, and presumably from the variability estimates provided for the raw data in the extended files they were consistent across animals. Nonetheless, was histological verification performed, confirming placement?

Some of the text is a little difficult to follow - sentence structure convoluted and could perhaps be made clearer. Similarly in Figure legends some revisions would increase clarity. A few examples are:

Pg 9 line 5 - PS increases in females but reach a ceiling effect, while PS values in aged males aroe 50% female PS values.

Pg 9 line 14 - increased in granule cell dendritic excitability - increase in or just increased?

Pg 10 line 5 - the three two three trains? Did you mean the first three trains?

Pg 11 line 14/Page 12 line 6. I am confused by what ‘sparse spatial representations’ and ‘predicted to be less sparse but possibly more robust’ means in these statements. Is less sparse mean more and therefore that is why more robust?

Pg 12 line 10- add male to beginning of this sentence

Suggest adding sample sizes to Figure 1

Figure 2 Caption Line 8 - this sentence could be reworded to increase clarity

Extended Figure 1 Caption- line 2 - add at baseline; end of line 6 add at baseline; perhaps point out variability in aged populations for EPSP slope? Line 7 add post-LTP When post LTP data were normalized

Assessment of NMDA component of evoked train response acknowledged investigations of Marin et al., 1992, but had been demonstrated by Racine et al, Brain Res 1991 556: 226-239 in freely moving rats and again by Joy and Albertson, Brain Res 1993 604: 273-282 in urethane anesthetized rat. Also paper by Burdette and Gilbert, Brain Res 1995 680: 53-62 reports on stimulus dependent effects on paired pulse depression at short and long inter-stimulus intervals in awake rat, largely consistent with those reported here albeit at a slightly longer interval of 250ms

The inclusion of extended data with submission is very integral to the study and allows experimenters to directly compare their findings at a more basic level that is not possible with the computations applied to the summarized data presented within the manuscript. I found the Extended data very useful in evaluating this manuscript. However I was not sure I had access to all the extended files - Labelling went from 1-4 and i found only 3 in the downloads.

## Author Response

Reviewer 1

The manuscript seeks to understand differences in plasticity at the medial perforant path-Dentate Gyrus synapses among sex and aging in rats. Changes in excitability, rather than the more classical synaptic changes, are reported by the authors in response to a relatively mild stimulation protocol.

The authors describe those changes in adult and aged males and females and investigate possible mechanisms for such differences. Changes in excitability are relatively poorly understood compared to synaptic plasticity. However, neural excitability contribution to learning, memory and pathological conditions is critical. The manuscript is, in general, well-written and follows a logical argument. The results are exciting. However, I have some comments:

1) It is not convincing to me to correlate the initial level of EPSP potentiation with the PS potentiation at all three time points considered (Figure 3). In my opinion, just the correlation for PS-EPSP baseline and PS-EPSP for the first 30min should be shown. If the 60min or 90min are shown, then the correlation should be done between the PS and EPSP corresponding to those timepoints.

• We would like to thank the reviewer for requesting the additional E-S analysis be performed using EPSP slope measures at parallel timepoints to the PS changes, rather than early EPSP slope only. As the potentiation of E-S slope after LTP tetani is transient across all groups, we first examined the E-S coupling relationship at the early EPSP slope period (0-30min post-LTP) as predictor of long-term changes in PS amplitude (0-30, 30-60, and 60-90min post-LTP). We now are presenting this data analyzed both ways (Figure 3 and Extended Material 3-1) as we believe it provides a more comprehensive understanding of the MPP dentate gyrus E-S coupling story contrasted by age and sex.

• We find the two methods of analyses (early EPSP slope versus parallel EPSP slope epochs) yields similar results in that it is a phenomenon is still restricted to female rats only, however, the analysis examining the early increases in EPSP slope more fully accounts for PS changes at later time periods in female rats of both ages (please see data in 5-9mo old females in Extended Material Figure 3-1B, parallel epochs, for the exception).

2) The heading “Increases in the population spike post-LTP in female rats depend on increases in EPSP slope” is misleading. According to Extended Data Figure 2-1, EPSPs are only transiently potentiated in all cases. Thus, EPSP potentiation cannot explain PS changes observed in aged females.

We would like to thank the reviewer again for this observation. Given that we include the second E-S coupling analysis (please see above), we have altered the heading to refer to the early increases EPSP slope increases specifically. The heading now reads: “Increases in the 2 2 population spike post-LTP in female rats are most predicted by early (first 30min post-LTP) increases in EPSP slope.”

3) a) Even though their information is implicitly shown in Fig.2, showing the I-O curves of PS and EPSP vs. Stimulation Intensity would help.

o We now include four new standard I-O graphs in Figure 2 (both the absolute and normalized data for both PS amplitude and the EPSP slope variables b) Also, error bars should be displayed in Figure 2.

• Thank you, this has been included now. Please note that error bars in normalized E-S coupling graphs (Figure 2F) are too small to be visible, but have been included. In this regard, even though at the test stimulation intensity used for baseline and subsequent recordings after tetanic stimulation (50% of maximum PS) no EPSP potentiation was shown (Extended Data Figure 2-1), it seems from some of the graphs (Figure 2) that EPSP values were larger post-tetanus than in baseline in some cases (for instance in Figure 2A5-9mo).

• We especially thank the reviewer on this observation. On review it was found that there had been an error in the generation of the original graph illustrating this point (old Figure 2; E-S normalized data, original submission). The correct graph is now shown in Figure 2F and is congruent with the data represented in Extended Material Figure 2-1. However, I have also some concerns about the I-O curves generated with the paired pulses every 10 secs and how this relatively short inter-stimulation interval and the paired pulses might have some effects on the I-O P1 values.

• Similar I-O and E-S profiles were reported in Walling and Harley (2004) in awake male rats using I-O current stimulation (0.1Hz; 100-1000uA) without paired pulse assay suggesting that paired pulse protocols performed at 0.1Hz, did not alter the I-O first pulse data, or E-S relationships.

4) The explanations are, in some instances, difficult to follow. For example, in the second paragraph of the Results section, it might help to include the particular graph the authors are referring to (i.e., PS values in aged males are ∼50% of female PS values (Figure 2A 18-20mo)).

• We have attempted to make this observation clearer for the readers. More specifically we have expanded the range (40-50%) as we believe this is a more accurate description.

5) Part of the Extended Data Figure 2-1 legend seems missing. It is not clear to me what is shown in panel B.

• Thank you, we have greatly expanded the description in the Figure Caption for Extended Data Figure 2-1.

6) Using Fisher LSD as a post-hoc test might have some drawbacks as it usually does not account for multiple comparisons.

• We have changed the post-hoc tests from Fisher’s LSD to Tukey HSD which included corrections for multiple comparisons.

3

3

7) The y-axis label on Figure 2B18-20mo should be “Population Spike (% of baseline)”.

• Thank you, this has now been corrected

8) How frequent was the test stimulation during baseline and following tetanic stimulation?

• This information is now included in both the graphic Figure 1, in the Figure Caption and in the Methods.

Reviewer 2

Beautiful comprehensive in vivo paper of short- and long-term plasticity measures in adult rats.

Elegant study design and large consistent field potential recordings. Topic areas has not been systematically addressed despite the significance of the model since its discovery and report over 50 years ago and the impact that discovery has had on the field. The analysis in this report is thorough and data reporting in main and supplemental materials, although dense, the graphics are complex but clear and complete.

Text on figures is a little on the small side in several of the figures.

• Text on all figures has been increased, where possible Some information on the software/hardware used to collect electrophysiological data could be included.

• In addition to the information on data acquisition and analysis software (SciWorks v9 or 11), we now also include source information for the constant current stimulation unit and the amplification and high-low pass filters. I did not see Extended Figures 2 or 3 in the materials. Extended Figure 1 was not labelled as such but I assume Temporal Profile is Extended Figure 1. Is there an Extended Figure 3 as the next Item listed is Extended Figure 4.

• Our apologies for any issues that may have occurred with figure identification. The original submission included three figures in the Extended Material section. The current resubmission now includes four extended figures. These are labelled 2-1, 3-1, 4-1, and 5-1 to correspond with the in-text figure to which it is referring. Modulating factors pertinent to the inquiry of sex and age differences that seem pertinent to the topic area but were not addressed by the authors include estrogen/estrous cycle in younger females and age-dependent differences in dentate gyrus neurogenesis.

• We now include a full paragraph (see page 13, line 278; corrected article) covering this query and the next query by the reviewer.

The final paragraph concludes that males may be more vulnerable to the aging-related disruption of plasticity. Although not investigated here it seems that some speculation could be offered by the authors on the role of estrogen in offered some degree of protection to females.

4

4

• We now highlight the potential protective role of estrogen in aging and dentate gyrus plasticity and further state that more work needs to directed to the understanding the role of sex hormones in normal aging. Please see new paragraph; page 13, line 278.

As noted by the authors, age-dependent behavioral impairments reported by work of Gallagher demonstrates a biomodal distribution of impaired and non-impaired aged rats. The variability observed in Extended Figure Temporal Profile of the present study certainly suggests a nonhomogeneous populations as well for electrophysiological attributes, especially for the EPSP (Figure B). Similarly, in the Extended Material Figure 5-1 could the more variable responses to old females at all lSI at the lower stimulus intensities, most notably post LTP, also reflect heterogeneity in the aging profile?

• We have placed a new comment in Discussion reflecting the identified heterogeneity of response. We thank the reviewer for emphasizing the relevance of heterogeneity amongst the age- and sex dependent responses. Please see page 14, line 297, corrected article)

Other Points

Curious to know if the authors experienced any additional difficulty with placement in the steretotaxic to attain a flat skull and consistency of electrode placement in the older animals relative to the young. Thicker skulls and alterations in toothbar angles can make the older animals more challenging. Perhaps this was mitigated somewhat from food restriction imposed.

• In part the food restriction was introduced to help keep a healthy aging profile for both male and female rats. We do find that the Sprague Dawley strain is extremely susceptible to obesity if left on ad libitum diets (in contrast to e.g. Long Evans). Thickness of skull, although prominent in aged animals, males particularly, did not impede electrode placements as brain surface with dura intact was used for initial dorsal/ventral (D/V) measures and the final D/V electrode placements were optimized by waveform visualization. The surgical procedures were performed by a single researcher who has extensive experience in in vivo MPP-DG recording. With experience, A/P adjustment can be made reliably for larger rats using the skull plate suture lines and length of skull. Some adjustments were made for size (and are now better reflected in the Methods description), but rarely were adjustments more than 100-300 um in the A/P and M/L plane.

It is clear from waveform morphology at the appropriate sites were hit, and presumably from the variability estimates provided for the raw data in the extended files they were consistent across animals. Nonetheless, was histological verification performed, confirming placement?

• Samples of electrode placements in Nissl-stained sections chosen from rats from each age and sex, have been added to Extended Material Figure 2-1A. Some of the text is a little difficult to follow - sentence structure convoluted and could perhaps be made clearer. Similarly in Figure legends some revisions would increase clarity. A few examples are:

• Pg 9 line 5 - PS increases in females but reach a ceiling effect, while PS values in aged males aroe 50% female PS values. 5 5

o This paragraph has been substantially modified to better communicate the smaller absolute (mV) PS values in male than female rats.

• Pg 9 line 14 - increased in granule cell dendritic excitability - increase in or just increased?

o Thank you. This should be ‘increase in’- this has been corrected

• Pg 10 line 5 - the three two three trains? Did you mean the first three trains?

o This should be ‘first three’ as indicated by reviewer- Thank you, this has been corrected.

• Pg 11 line 14/Page 12 line 6. I am confused by what ‘sparse spatial representations’ and ‘predicted to be less sparse but possibly more robust’ means in these statements. Is less sparse mean more and therefore that is why more robust?

o We now replace the phrase in question with “MPP-supported spatial and nonspatial representations might be predicted to be less diffusely distributed, and therefore possibly more robust, in female rats than males”;

• Pg 12 line 10- add male to beginning of this sentence

o Corrected - thank you

• Suggest adding sample sizes to Figure 1

o This information has now been included this information diagrammatically in Figure 1, as requested.

• Figure 2 Caption Line 8 - this sentence could be reworded to increase clarity

o Thank you we agree that this sentence should have been worded more clearly. We have consequently split this sentence into two and reworded for clarity (changes marked in red in Figure Caption)

• Extended Figure 1 Caption- line 2 - add at baseline; end of line 6 add at baseline; perhaps point out variability in aged populations for EPSP slope? Line 7 add post-LTP When post LTP data were normalized

o Thank you, this has been corrected

• Assessment of NMDA component of evoked train response acknowledged investigations of Marin et al., 1992, but had been demonstrated by Racine et al, Brain Res 1991 556: 226-239 in freely moving rats and again by Joy and Albertson, Brain Res 1993 604: 273-282 in urethane anesthetized rat. Also paper by Burdette and Gilbert, Brain Res 1995 680: 53-62 reports on stimulus dependent effects on paired pulse depression at short and long inter-stimulus intervals in awake rat, largely consistent with those reported here albeit at a slightly longer interval of 250ms.

o We now include reference to these studies within the manuscript and though the methods are not identical to the ones used here (e.g. Racine’s analysis on single pulse evoked responses versus Maren’s analysis on pulse trains) we have incorporated these 6 6 with explanation within the text. We thank the reviewer for alerting us to the Burdette and Gilbert study and the congruencies with the results reported here. We now include reference within the Figure Caption of Extended Material Figure 5-1

• The inclusion of extended data with submission is very integral to the study and allows experimenters to directly compare their findings at a more basic level that is not possible with the computations applied to the summarized data presented within the manuscript. I found the Extended data very useful in evaluating this manuscript. However I was not sure I had access to all the extended files - Labelling went from 1-4 and I found only 3 in the downloads. o In this resubmission, we now include four figures in the Extended Material (labelled Fig. 2-1, 3-1, 4-1 and 5-1 to correspond to the appropriate Figure from the main body of the manuscript.
