# Supplementary figures and images for: Sprague-Dawley Rats Differ in Responses to Medial Perforant Path Paired Pulse and Tetanic Activation as a Function of Sex and Age
Source: eNeuro. 2023 Jul 4;10(7):ENEURO.0431-22.2023. doi: 10.1523/ENEURO.0431-22.2023 (PMC10327532; doi:10.1523/ENEURO.0431-22.2023)

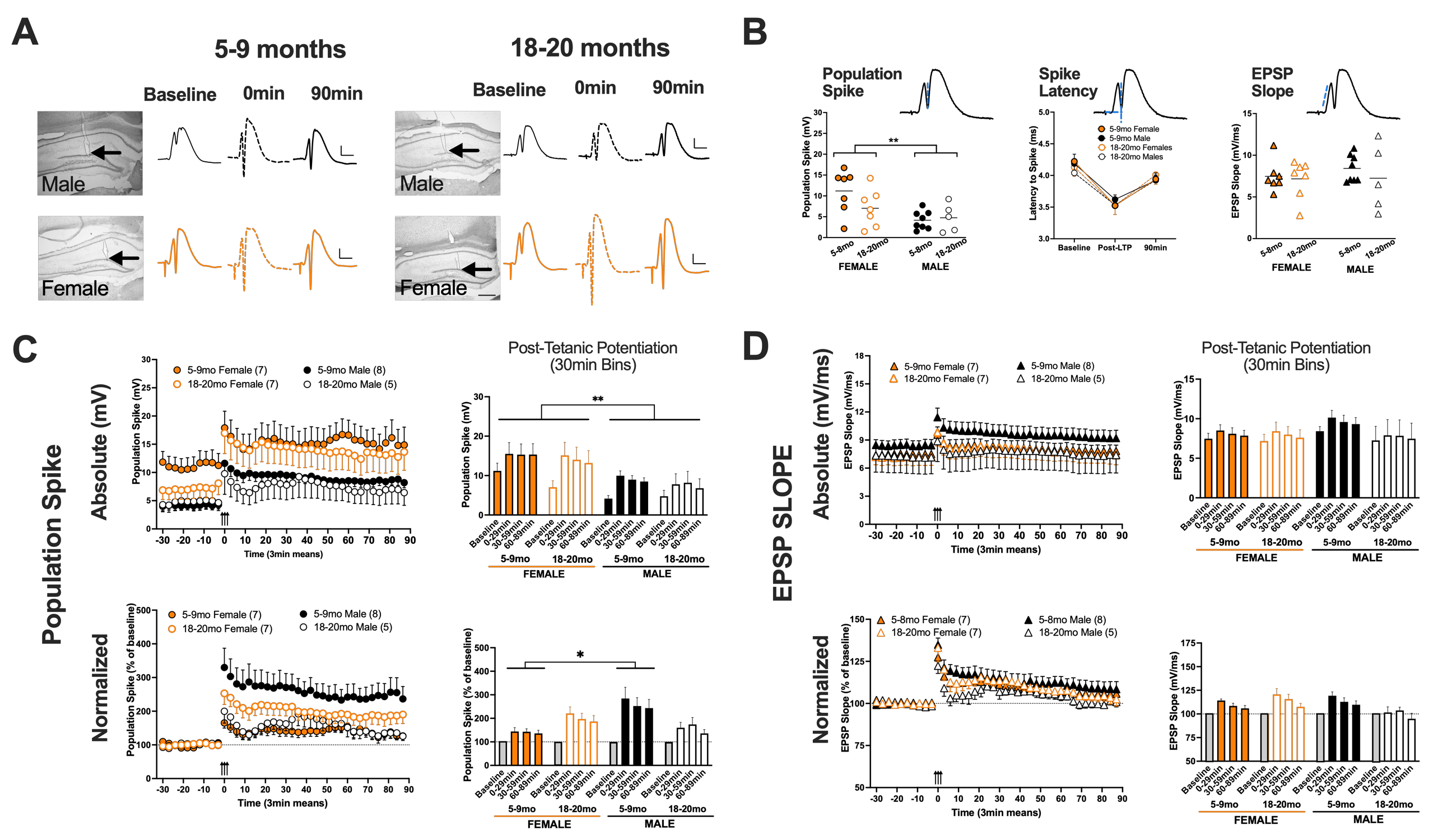

Supplement: Extended Data Figure 2-1 — The effects of a moderate strength tetanic LTP protocol on the perforant path-dentate gyrus evoked population spike (PS) and EPSP slope measures in adult (5–9 months) and aged adult (18–20 months) urethane-anesthetized male and female rats. A, Sample dentate gyrus recording electrode placements and MPP-dentate gyrus evoked potential waveforms for three periods before (baseline) and after (0 and 90 min) moderate strength tetanic stimulation. Scale bar is 500 μm in micrograph and 4 mV and 5 ms in waveforms. B, Absolute PS amplitude (baseline current level), PS latency (baseline, 0 min, and 90 min post-LTP) and EPSP slope measures (baseline current level). PS values (mV) were significantly higher in female rats compared to male rats at baseline current levels (first graph). PS latency decreased similarly post-LTP for all groups (middle graph). Absolute EPSP slope values did not differ between groups at baseline current intensity (third graph). C, Temporal profile (X-Y plot), and 30-min binned data (bar graph) of absolute (top panels), and normalized (bottom panels) PS data. Female rats had significantly larger PS (mV) amplitude measures than male rats (main effect sex, F(1,23) = 7.378; p = 0.012). Normalization of PS data illustrates adult males (5–9 months) had higher percentage PS increases than aged males (18–20 months), and adult female rats (age, sex, LTP interaction, F(3,69) = 6.52; p = 0.0006, with post hoc). D, EPSP Slope data. No sex-dependent or age-dependent differences were observed in absolute EPSP slope measures (top panels). When post-LTP EPSP slope values were normalized to baseline measures; however, a significant age × sex interaction was also revealed (F(1,23) = 5.703; p = 0.026). Normalized baseline measures were not included in the statistical analyses in C or D (gray bars). * minimum p < 0.05, **p < 0.01. Download Figure 2-1, TIF file. [file enu-eN-NWR-0431-22-s02.tif]

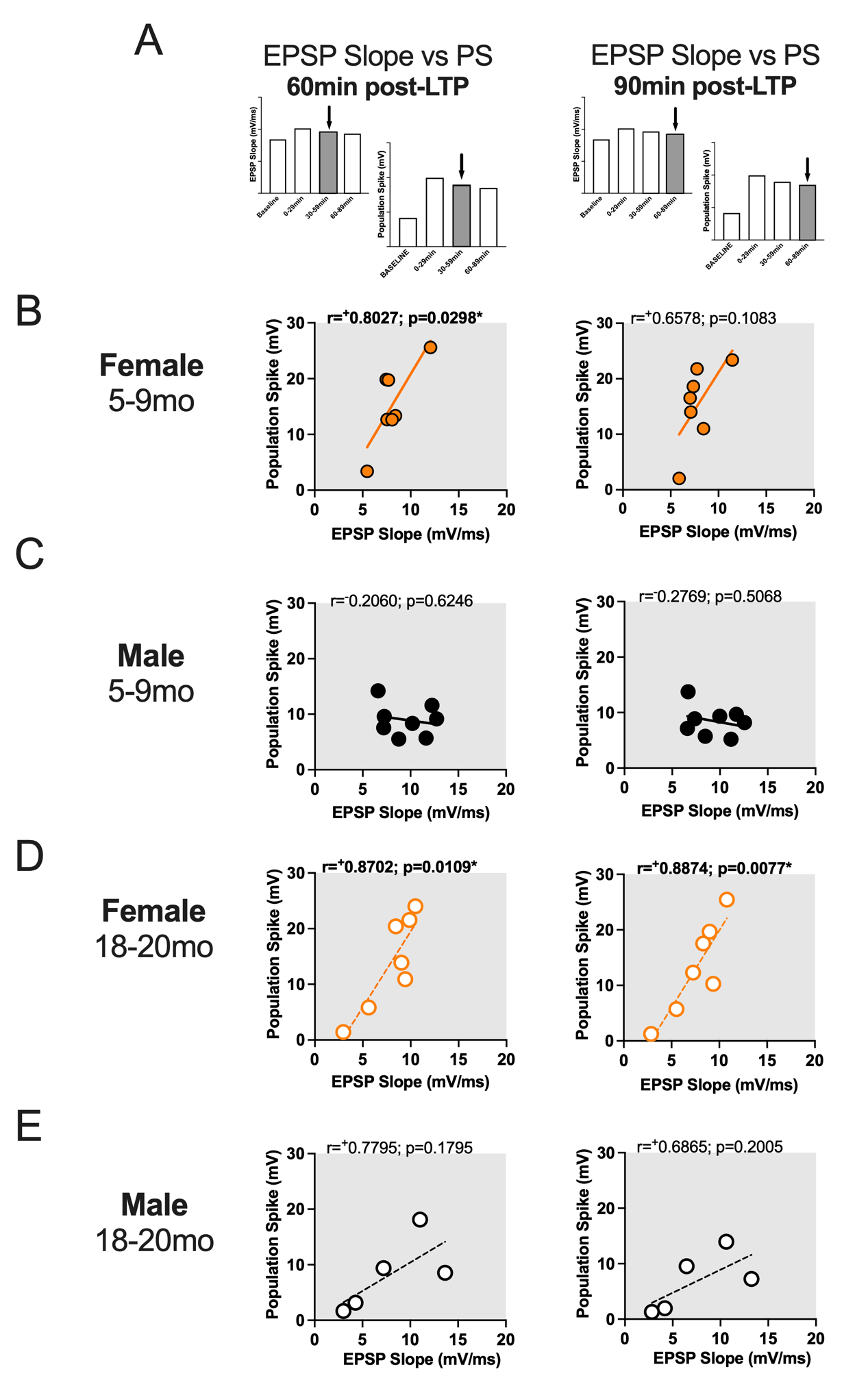

Supplement: Extended Data Figure 3-1 — The effects of a moderate strength tetanic LTP protocol on the perforant path-dentate gyrus evoked population spike in young (5–9 months) and old (18–20 months) urethane-anesthetized male and female rats examined at matched 30- to 59- and 60- to 90-min time periods. Similar to results examining the correlation between early (0–30 min) post-LTP EPSP slope potentiation on population spike potentiation at 0–30, 30–60, and 60–90 min post-LTP periods (presented in Fig. 3), the EPSP slope potentiation of female rats (adult and aged adult) was still most often a predictor of PS potentiation; however in contrast to the early EPSP potentiation (Fig. 3), late (60–90 min) EPSP slope potentiation did not significantly correlate with PS potentiation in adult (5–9 months) females. Male rat EPSP slope potentiation (early or late) did not correlate with PS potentiation at any of the post-LTP periods. Download Figure 3-1, TIF file. [file enu-eN-NWR-0431-22-s03.tif]

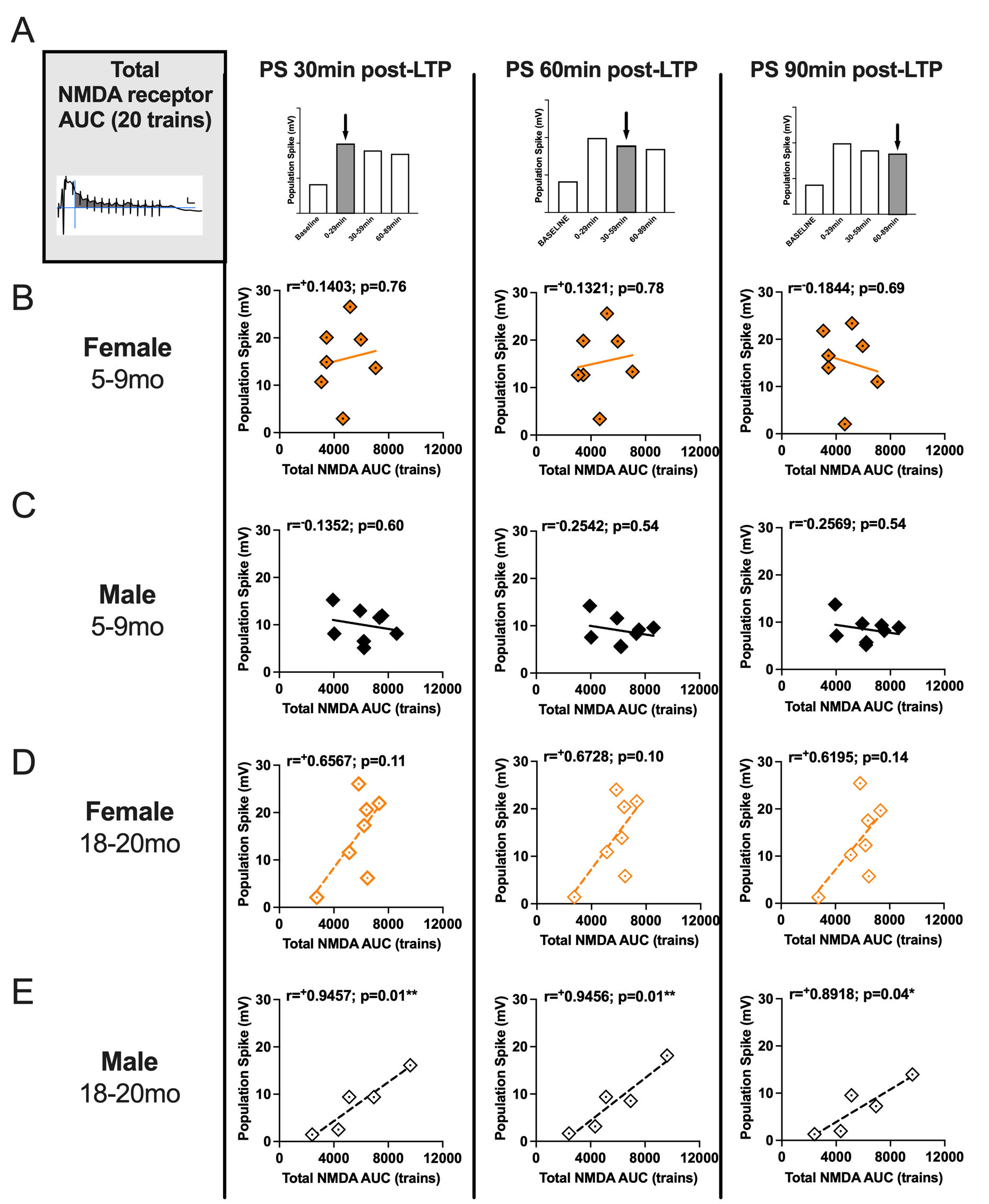

Supplement: Extended Data Figure 4-1 — Total NMDA receptor contribution during moderate tetanic LTP stimulation correlated with population spike increase in 30min bins in adult and aged male and female rats. The total NMDA receptor Area Under the Curve (AUC) for the 15 pulse, and 20 tetanic trains was plotted against the absolute PS amplitude (mV) for the post-tetanic period (0-90min post-LTP). Total NMDA AUC was not correlated with absolute PS values (A-B) however, a significant correlation was more associated with aged male (18-20mo, see E), and trend in aged female rats (D). Download Figure 4-1, TIF file. [file enu-eN-NWR-0431-22-s04.tif]

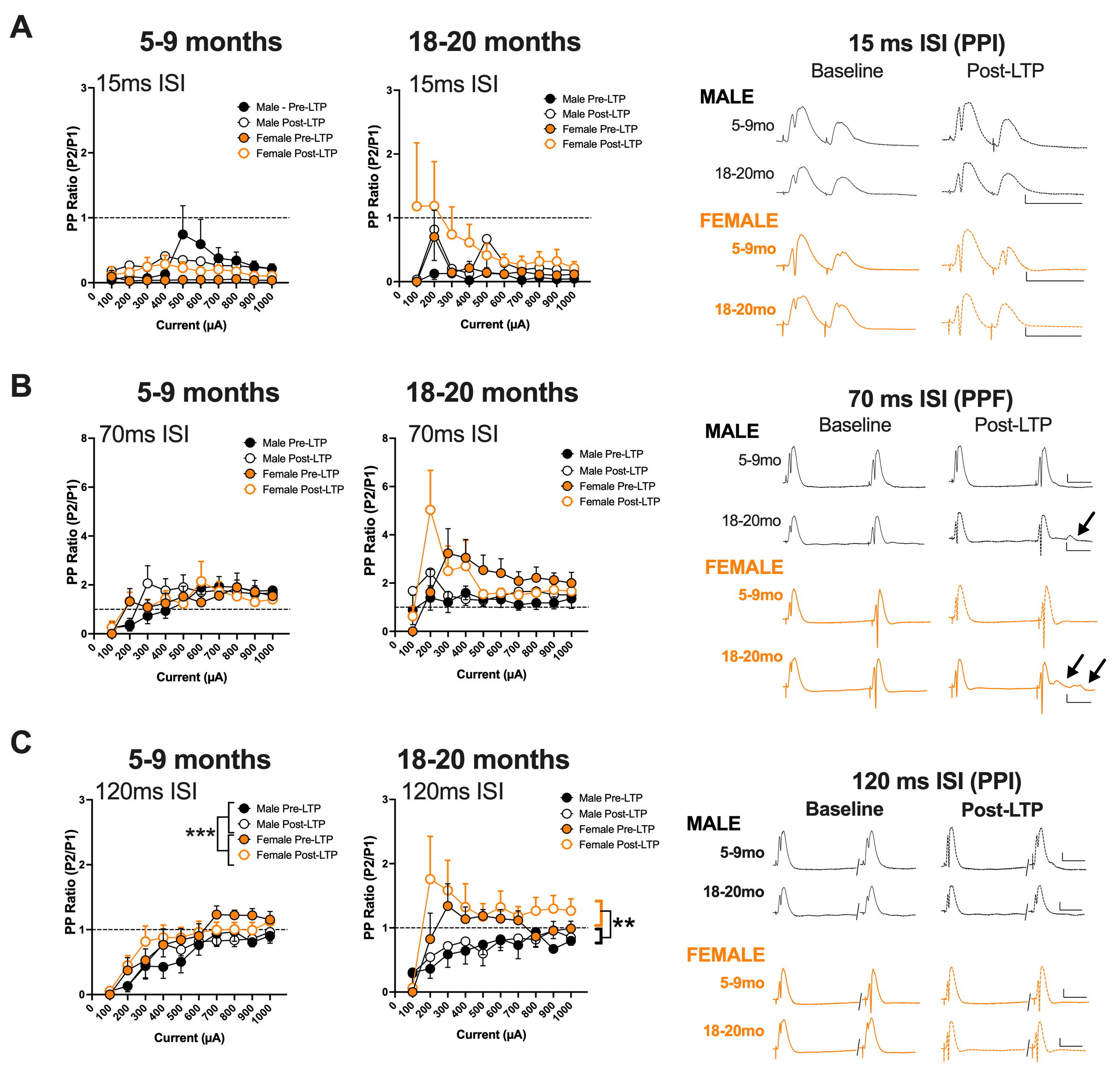

Supplement: Extended Data Figure 5-1 — The effects of a moderate tetanic LTP protocol on paired pulse ratio input-output current intensity curves for male and female adult and aged adult Sprague Dawley rats at three interstimulus intervals. A, 15-ms ISI (PPI); no significant main effects or interactions of sex for either age groups were observed. B, 70-ms ISI (NMDA-sensitive PPF; Joy and Albertson, 1993) no significant main effects or interactions of sex for either age groups, however examples of hyperexcitability emerged in some male and female rats (examples shown, arrows in waveforms). These could not be quantified. C, 120-ms ISI (PPI). In the five- to nine-month age group, there was a significant main effect of Age (F(1,13) = 18.46; p < 0.001), and in the 18- to 20-month rats, a significant current × sex interaction (F(9,90) = 3.106; p = 0.003). Burdette and Gilbert (1995) report late PPI at low current levels (200-ms ISI) in behaving male Long–Evans rats similar to the urethane-anesthetized adult (5–9 months) male and female and aged male Sprague Dawley rats here; however, this profile was less observed in aged adult (18–20 months) female rats in the present study. Data represent means ± SEM; **p < 0.01 and ***p < 0.001. Scale in waveforms is 4 mV/20 ms. Download Figure 5-1, TIF file. [file enu-eN-NWR-0431-22-s05.tif]
